# Global basin-scale mapping of pH and alkalinity in inland waters

**DOI:** 10.1038/s41597-026-07028-2

**Published:** 2026-03-14

**Authors:** Meritxell Batalla, Jordi Martínez-Artero, Jordi Catalan

**Affiliations:** 1 CREAF, Environmental Change Ecology Group (GECA), Cerdanyola del Vallès, Catalonia Spain; 2 Spanish National Research Council, CSIC, Bellaterra, Barcelona E-08193 Spain

**Keywords:** Carbon cycle, Limnology, Geochemistry, Freshwater ecology, Carbon cycle

## Abstract

The acidity and buffering capacity of inland waters are essential for biogeochemical processes and impose significant constraints on the distribution of freshwater species. Although many measurements exist worldwide, the data distribution is biased toward more-studied regions, and a global assessment of gradients and their spatial distribution is lacking. In the PHALK dataset, we compile alkalinity and pH values for continental surface waters worldwide, collating chemical data from 18 source databases and 55 scientific publications. A quality-control filter yielded high-quality alkalinity and pH datasets, including 50,916 and 107,896 sites, respectively. Based on the collated dataset and a random forest model, pH and alkalinity in surface waters were modeled worldwide at the basin scale (HydroBASINS v1 sub-basin level 12: 1,034,083 drainage basins) using 23 variables describing basin geological and hydrological characteristics. Each extrapolated value is accompanied by two uncertainty indicators: environmental differentiation, based on the similarity of the basin’s environmental conditions to those of basins with measured data, and upscaling confidence, based on the variation in the random forest’s internal bootstrap.

## Background & Summary

The colonization of the inland aquatic environment by life has faced greater chemical variation than in the marine environment. The new ecophysiological challenges have been an opportunity for functional and taxonomic diversification^1–3^, and currently, this chemical variation is still part of the main ecological and evolutionary processes^4^. A large part of the chemical variation in inland waters is related to pH-alkalinity gradients^5^. The bedrock nature and its weathering rate^6^ eventually provide the dissolved salts that determine the balance between strong anions and cations, which ultimately influence the inorganic carbon that can be dissolved in equilibrium with atmospheric CO_2_ and the buffering capacity for acids (total alkalinity). The relationship between pH and alkalinity is semilogarithmic^7^, and deviations depend on weak acid concentrations and exposure to the atmosphere^8^. Generally, pH gradients are relevant for species segregation and evolutionary challenges at their extremes^9–11^. Despite the interest in chemical and biological studies of inland waters, a worldwide estimate of pH and alkalinity distributions was lacking. Some continents or regions within them have abundant data, and others are markedly understudied. Recent initiatives have compiled and harmonized water chemistry datasets, such as The Global River Water Quality Archive (GRQA)^12^, with river data, and the Surface Water Chemistry (SWatCh) database^13^, with lake, river, and reservoir data. Both databases have code and data openly accessible on GitHub and Zenodo. Building on GRQA^12^ methods, we developed a new dataset with expanded spatial coverage, focusing on alkalinity and pH, using 18 datasets from existing source databases and 55 publications (Table 1). Furthermore, we filtered the alkalinity data, applying data quality assurance based on ionic balance. Finally, based on catchment characteristics, we scaled up the data worldwide to predict pH and alkalinity for drainage basins.

**Table 1:** Repositories and datasets used for the compilation of the hydrochemical dataset.

Dataset	Source name	Reference
*Large repositories*		
GLORICH	Global River Chemistry Database	Hartmann *et al*.^21^, Hartmann *et al*.^22^
GEMStat	The Global Freshwater Quality Database	United Nations Environment Programme 2018^19^
WATERBASE	Waterbase – Water Quality	European Environment Agency 2019^23^
CESI	Canadian Environmental Sustainability Indicators (Water quality in Canadian rivers)	Environment and Climate Change Canada 2019^24^
WQP	The Water Quality Portal	Read *et al*.^25^, United States Geological Survey 2022^26^
*Regional datasets*		
ESTONIA	Short-term high-frequency water dissolved carbon dioxide, temperature, dissolved oxygen, salinity, and pH data from 8 Estonian lakes in the year 2014	Laas & Khan 2019^49^
MYVATN	LTREB Chemical and Physical Limnology at Lake Myvatn 2012-current	Ives *et al*.^50^
TSADB	Tropical South America Diatom and Waterbodies database	Benito 2021^51^
NADUF	National long-term surveillance of Swiss rivers	Eawag & FOEN 2020^52^
CDD	Circumpolar Diatom Database	Pienitz & Cournoyer 2017^53^
MONGOLIA	Limnological Catalogue of Mongolian Lakes	Alonso 2010^54^
CWSM	An inorganic water chemistry dataset of rivers, dams, and lakes in South Africa	Huizenga *et al*.^55^
AKROTIRI	Water chemistry data from Lake Akrotiri, Cyprus, and its main inputs, July 2019 - November 2020	Bowes *et al*.^56^
ARCTIC	Nutrient chemistry of Arctic Lakes in Greenland, Norway, Russia, and Alaska	Whiteford *et al*.^57^
BEL&UKR	Water chemistry of seven lakes in Belarus and Ukraine, 2014 to 2016	Lerebours & Smith 2019^58^
GEM	The Greenland Ecosystem Monitoring (GEM) Database	Greenland Ecosystem Monitoring Program 2020^59,60^
NSLS	Nova Scotia Lake Chemistry Data	Government of Nova Scotia 2019^61^
Other data	Other data	Authors’ group research data
*Data from scientific literature*		
LITERATURE	Scientific papers and reports	23 concerning alkalinity^62–83^ and 54 concerning pH^62–82,84–115^

The global upscaling of alkalinity and pH in inland waters was based on previous studies that identified key environmental characteristics that determine water chemistry in drainage basins^14–17^. The majority of those studies considered the regional scale; only a few attempted a worldwide approach. Lehmann *et al*.^17^ conducted a global spatial study, although only 233 sites were included in the analysis. Marcè *et al*.^18^ generated a global map of alkalinity with 101 sites sampled in the study and 584 stations from GEMStat^19^ to validate the model, with runoff and lithological variables as drivers. Our scaling-up was based on 50,916 high-quality data alkalinity sites and 107,896 pH sites globally distributed, including high spatial and environmental heterogeneity, and modeled using 23 environmental basin characteristics and extrapolated to 1,034,083 drainage basins defined by HydroBASINS v1^20^.

## Methods

### Dataset sources

Global water chemistry data were compiled for inland waters (rivers, lakes/reservoirs, and wetlands) from May to July 2022. Data sources included repositories, files provided in the supporting materials of scientific publications, and data extracted from the main text of scientific articles and reports. The focus was on pH and alkalinity, but accompanying variables such as conductivity, cations (calcium, magnesium, potassium, sodium, and ammonium), and anions (chloride, nitrate oxides, and sulfate) were also collated for the quality assurance procedure (see below).

**Fig. 1:**
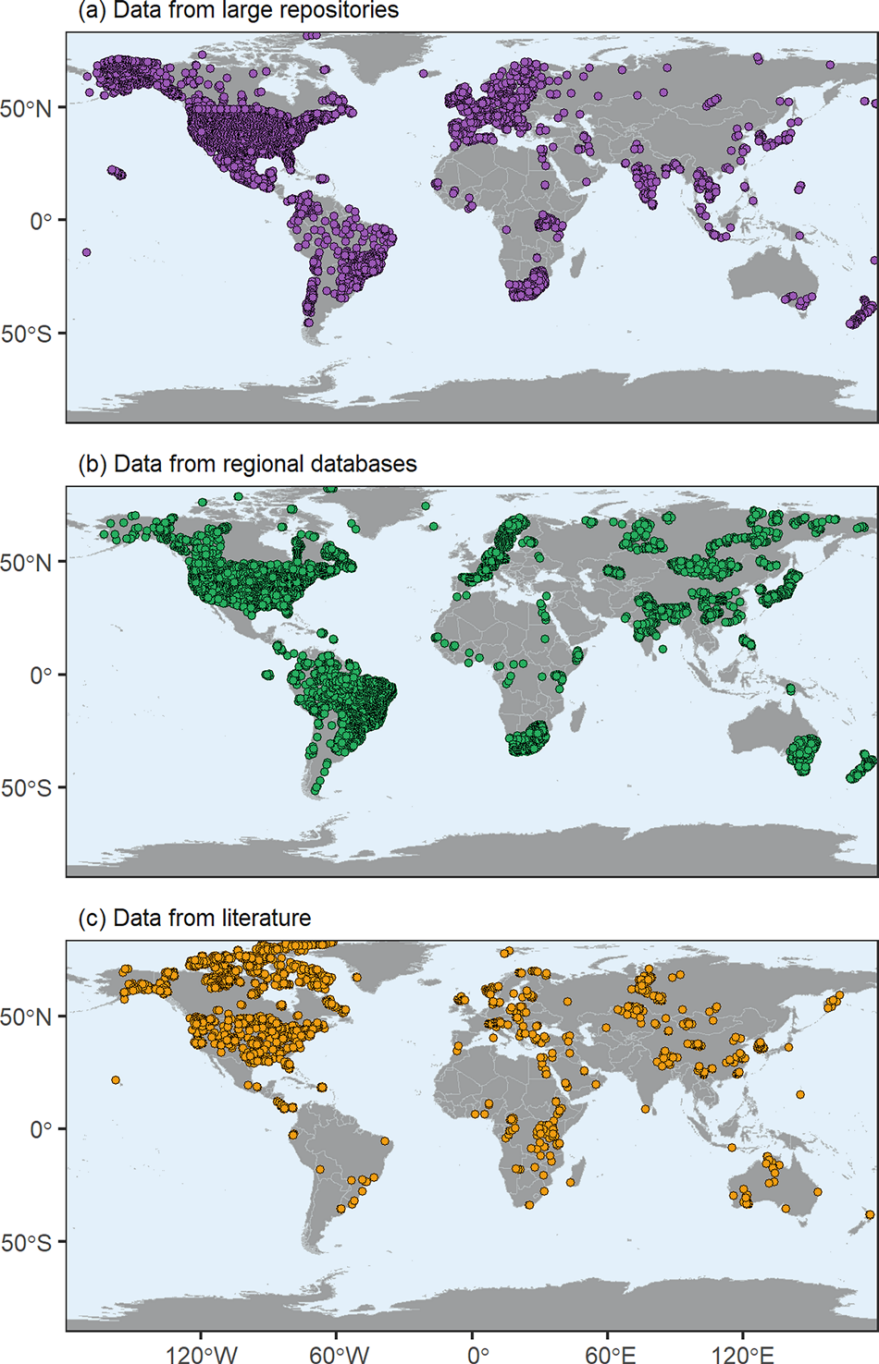
Spatial distribution of the hydrochemical field data collected from (**a**) large repositories, (**b**) regional databases, (**c**) literature sources.

Five repositories provided a large amount of data and observation sites around the world (Table 1): two global databases, GLObal RIver Chemistry (GLORICH)^21,22^ and Global Freshwater Quality Database (GEMStat)^19^; one European database, WATERBASE^23^; and two national databases, the Canadian Environmental Sustainability Indicators program (CESI)^24^ and the Water Quality Portal (WQP)^25,26^ from the US. Nevertheless, the sites were markedly biased towards the most studied regions of the world (Fig. 1a). Consequently, an effort was made to fill the least represented world areas (Table 1) with data extracted from regional repositories (Fig. 1b) and bibliography (Fig. 1c). Only data with explicit georeferencing or associated with maps of acceptable coordinate resolution were used. For some lake data lacking coordinates, a search by lake name was performed, and the results were georeferenced if no ambiguity existed.

### Dataset preparation

The dataset preparation involved data preprocessing and quality control of alkalinity; the details are provided in two subsections. Briefly, each source dataset was first preprocessed to select the variables of interest and harmonize data formats. We followed Virro *et al*.^12^ for the harmonization and filtering of the hydrochemical data, using Python scripts available at https://github.com/LandscapeGeoinformatics/GRQA_src^27^ and adapting them to our needs regarding water body type, targeted chemical variables, and data sources (Table 2). Second, the data were summarized for sites with multiple measurements over time using the median to represent each site. Thereafter, all datasets were merged by variable, followed by the identification and removal of duplicate records across sources. Finally, an alkalinity quality control was applied to detect potentially inaccurate measurements.

**Table 2:** Comparison of the GRQA^12^ dataset and the data collected for this study.

	GRQA	This study
**Water bodies**	Rivers	Rivers, Lakes/Reservoirs, Wetlands
**Variables**	Nutrients, Carbon, Sediments, Oxygen	Alkalinity, Ammonium, Calcium, Chloride, Conductivity, Magnesium, Nitrate (i.e., Nitrate, Nitrate + Nitrite, Oxidized nitrogen), pH, Potassium, Sodium, Sulfate
**Data source**	CESI^24^, GEMStat^19^, WQP^25,26^, GLORICH^20,21^, WATERBASE^23^	See Table 1

#### Preprocessing

Data downloading was automated when possible, particularly for large repositories (CESI^24^, GLORICH^20,21^, WATERBASE^23^, and WQP^25,26^). For GEMStat^19^, a custom request had to be submitted to the data portal. Data, either automatically or manually downloaded, was placed in a folder for each source. Because each data source contained different variable names, codes, units, and chemical forms, we generated a code file for each data source following the structure in Virro *et al*.^12^. The file contained all the information necessary to process the data homogeneously, including variable codes, the original units of the data, and conversion factors to harmonize the units (i.e., alkalinity in µeq/L, conductivity in µS/cm, and ions in mg/L). For CESI^24^ and WQP^25,26^, a coordinate conversion was needed because the original site coordinates were referenced to the North American Datum of 1983 (NAD83). They were converted to the World Geodetic System 1984 (WGS84), which was used in the other data sources.

An initial data filtering was made to detect and delete records from sites with missing coordinates or spatially inconsistent information. Then, all values equal to or lower than zero were removed except for alkalinity, whose negative values may be meaningful. Some datasets contained information about the detection limits for the measured ions. In those cases, data below the detection limit (b.d.l.) were substituted by multiplying the detection limit by 0.1, without compromising the ionic balance performed in a later step of quality control. Some datasets include a quality check flag, and values flagged as low-quality were excluded. Finally, a file for each variable was generated for each data source, including each observation, site information, the sampling date (if available), and the variable values in harmonized units.

There was considerable variation in the number of observations per site, with some corresponding to long-term monitoring and others to occasional surveys. Because we were aiming to consider worldwide pH and alkalinity spatial gradients rather than temporal trends, we characterized each site by summarizing the observations, including the earliest and oldest year of the observations, the number of observations, and a statistical description (i.e., mean, median, 10% quantile, 90% quantile, and standard deviation).

The summarized site datasets from the distinct data sources were merged into a single dataset and filtered to avoid site duplicates using the following procedure^12^: (1) we clustered the sites within a 1 km radius of each other and calculated the root mean square error (RMSE) of pairs using the median of the summarized sites; (2) site pairs with a root mean square error (RMSE) of zero were flagged as potential duplicates, and a list of all such pairs was compiled; (3) if the records corresponded to a different water type (e.g., lake and stream), both records were kept, if not, the paired site with the lowest number of observations was deleted.

#### Alkalinity quality control

Although some data sources may have applied analytical quality controls to the collated chemical data, we performed a comprehensive quality control to evaluate potentially erroneous values. A single table was created that joined all individual chemical variable files. We divided the data into two groups: those with complete records of cations and anions for ionic balance calculation (13%) and those with some missing information (87%). For those sites with a complete record, we calculated three indicators of potential analytical or data processing errors: the percentage of ionic imbalance (IB), the percentual difference between ion-estimated and measured conductivities (Cond_dif_), and the percentual difference between estimated and measured alkalinity (Alk_dif_).

1 $${\rm{IB}}=100\times \frac{\sum [{\rm{cations}}]\,-\,\sum [{\rm{anions}}]}{\sum [{\rm{cations}}]\,+\,\sum [{\rm{anions}}]}$$

2 $${{\rm{Cond}}}_{{\rm{dif}}}=100\times \frac{\left({{{\rm{Cond}}}_{{\rm{meas}}}-{\rm{Cond}}}_{{\rm{est\_cor}}}\right)}{{{\rm{Cond}}}_{{\rm{est\_cor}}}}$$

where Cond_meas_ is the conductivity measured, and ${{\rm{Cond}}}_{{\rm{est\_cor}}}={{\rm{Cond}}}_{{\rm{est}}}\times {{\rm{IS}}}^{2}$, based on an expected conductivity calculation ${{\rm{Cond}}}_{{\rm{est}}}={\Sigma ({\rm{m}}}_{{\rm{i}}}\times {{\rm{\lambda }}}_{{\rm{i}}})/1000$, considering each ion concentration (m_i_, µeq/L), and its ionic molal conductivity (*λ*_*i*_) and the ionic strength, ${\rm{IS}}=\left(\sum {{\rm{m}}}_{{\rm{i}}}\times {{\rm{c}}}_{{\rm{i}}}\right)/1000/2000$, where c_i_ is the ion charge.

3 $${{\rm{Alk}}}_{{\rm{dif}}}=100\times \frac{\left({{\rm{Alk}}}_{{\rm{meas}}}-{{\rm{Alk}}}_{{\rm{est}}}\right)}{{{\rm{Alk}}}_{{\rm{est}}}}$$

where Alk_meas_ is the alkalinity measured, and ${{\rm{Alk}}}_{{\rm{est}}}=\sum [{\rm{conservative\; cations}}]\,-\,\sum [{\rm{conservative\; anions}}]$.

Some errors, apparently related to unit conversions, were detected. We found that for the GEMStat^19^ source, the India data, and part of the Mexico dataset, the estimated alkalinity systematically differed from the measured one in a way that suggested a unit conversion error in the calcium and magnesium concentrations, since dividing them by 2 (corresponding to their charge) and recalculating the estimated alkalinity, it agreed with the measured. For the GLORICH^20,21^ source, a systematic deviation was detected between measured and estimated conductivity in the Germany, South Africa, and Sweden datasets. The discrepancies were corrected by multiplying the measured conductivity by 100 in the Germany data and by 10 in the South Africa and Sweden data.

We established a threshold of *IB* = 5% to differentiate between high- and low-quality sites^28^, which resulted in 22,862 sites in the high-quality category. Additionally, the quality type was set to low for high-quality sites in which the absolute value of the estimated-to-measured alkalinity difference was 100% or greater. Only 529 sites accomplished this condition. We took this decision after exploring the relationship between alkalinity and pH and finding that at relatively low pH (<7), some large discrepancies between estimated and measured alkalinity corresponded to samples that deviated markedly from the random variation of the pH-log-alkalinity relationship. Therefore, despite an acceptable ionic balance, the accuracy of these data was suspicious.

**Fig. 2:**
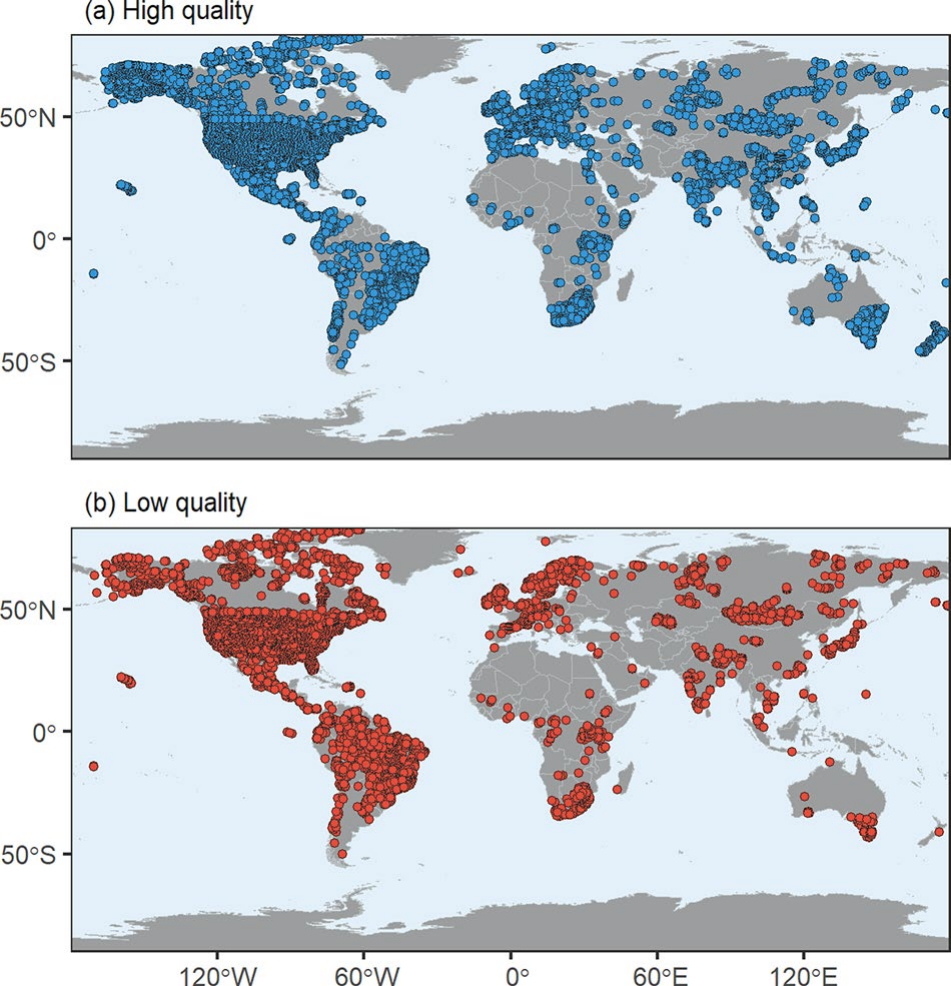
Alkalinity data quality distribution. High- (**a**) and low-quality data (**b**) were assigned using ionic balance or the Bayesian approach described in the text.

Finally, a Gaussian Naive Bayes (GNB) model was trained using a subset of the data for which quality could be assessed and classified, to categorize measurements for which only partial information was available regarding the quality assurance requirements described above. GNB is a probabilistic classification technique used in Machine learning, based on the normal distribution of the input variables and assuming feature independence^29^. It was applied with the naivebayes R package (version 1.0.0)^30^. Based on observed features, the model estimates the probability that a given sample belongs to a specific class (low- or high-quality). We used partial information on predictors, including cations, anions, pH, alkalinity, or conductivity. A prior distribution of 0.77 (high-quality) and 0.23 (low-quality) was established to reflect the known representativeness of each quality class in the dataset. The model was trained using 70% of the data, with the remaining 30% reserved for testing, achieving a classification accuracy of 78% on the test set. The model was then used to predict data quality for the remaining observations with incomplete information, predicting that 87% of the samples were high-quality and 13% were low-quality. This approach allowed us to extend the quality classification to the entire dataset while preserving spatial coverage (Fig. 2). The final alkalinity reference dataset used for upscaling was the high-quality subset, comprising 50,916 sites.

### Scaling up

#### Drainage basin variables

We considered several drivers of alkalinity and pH variation at the basin scale (Table 3), including environmental basin characteristics, catchment morphology, and the lithological composition of the bedrock. The HydroSHEDS v1 project^31^ provides hydrographic data products at a global scale derived from the Shuttle Radar Topography Mission (SRTM) digital elevation model at 3 arc-second resolution. HydroBASINS^20^, one of the products, provided the polygonal delineation of sub-basin boundaries, including 12 hierarchically nested sub-basin divisions. In this study, we used the more detailed level (12) as geographical units of the upscaling model, the distance to the most downstream hydrological sink (DIST_MAIN, usually a proxy for the distance to the coast), and the total upstream drainage basin area of the sub-basin considered. Another HydroSHEDS^31^ product, HydroATLAS^32,33^, was the data source for land surface runoff at the sub-basin scale, elevation, terrain slope, snow cover extent, forest cover extent, and annual average air temperature for the total upstream watershed of the sub-basin. HydroATLAS provides no data on Greenland’s terrain slopes. We used the digital elevation mosaics and an ice cover mask from the NASA Greenland Ice Sheet Mapping Project (GrIMP^34^) to select Greenland HydroATLAS basins without ice cover, and we calculated the average slope for each sub-basin.

**Table 3:** Independent variables used in the alkalinity and pH upscaling models.

Variable	Code	Source
Land surface runoff	run_mm_syr	HydroATLAS (HydroSHEDS)
Elevation	ele_mt_uav	HydroATLAS (HydroSHEDS)
Terrain slope	slp_dg_uav	*HydroATLAS (HydroSHEDS) + GrIMP
Snow cover extent	snw_pc_uyr	HydroATLAS (HydroSHEDS)
Forest cover extent	for_pc_use	HydroATLAS (HydroSHEDS)
Annual average air temperature	tmp_dc_uyr	HydroATLAS (HydroSHEDS)
Distance to the most downstream sink	DIST_MAIN	HydroBASINS (HydroSHEDS)
Total upstream area	UP_AREA	HydroBASINS (HydroSHEDS)
% of unconsolidated sediments	XX_su_U	*GLIM
% of siliciclastic sedimentary rocks	XX_ss_U	*GLIM
% of mixed sedimentary rocks	XX_sm_U	*GLIM
% of carbonate sedimentary rocks	XX_sc_U	*GLIM
% of pyroclastics	XX_py_U	*GLIM
% of evaporates	XX_ev_U	*GLIM
% of metamorphic rocks	XX_mt_U	*GLIM
% of acid plutonic rocks	XX_pa_U	*GLIM
% of intermediate plutonic rocks	XX_pi_U	*GLIM
% of basic plutonic rocks	XX_pb_U	*GLIM
% of acid volcanic rocks	XX_va_U	*GLIM
% of intermediate volcanic rocks	XX_vi_U	*GLIM
% of basic volcanic rocks	XX_vb_U	*GLIM
% of ice and glaciers	XX_ig_U	*GLIM
% of water bodies	XX_wb_U	*GLIM

All the variables correspond to the upstream watershed of the sub-basin considered, except the land surface runoff, which corresponds to the annual average within the sub-basin. * Variables with an asterisk were calculated in this project and provided in the final dataset.

Although HydroATLAS includes information on lithological classes from the Global Lithological Map (GLiM^35^), the simplified grid version at 30 arc-minute resolution was too coarse for our purpose. Therefore, we overlaid the basin polygon layers with the original GLiM vector map, yielding a high-resolution lithological description at a global scale. We considered the dominant lithological category and the percentage of each lithological category at sub-basin and upstream watershed resolutions. We used the first level of GLiM lithological categories, which considers 16 categories. The upstream watershed lithology ultimately showed greater explanatory power than the sub-basin lithology in modeling alkalinity and pH.

Collinearity between predictor variables was low due to the different nature of the variables and the global scale. Only air temperature and snow cover extent, with a Pearson correlation of –0.88, exceed the commonly used threshold of 0.7^36^. The correlation arises at a global scale because, at high air temperatures, snow does not occur. However, we kept both variables because in low-temperature areas they provide complementary information that could improve model predictions. Also, a variance inflation factor (VIF) analysis was applied to the predictors. It provides an index quantifying the increase in the variance explained of a variable in a regression due to multicollinearity. Only the lithological classes showed a high VIF due to the large number of coincident zeros. However, they were retained because they were complementary in terms of information content when they were non-zero^37^.

#### Watersheds with surface water

HydroATLAS used the information from the Global Lakes and Wetlands Database (GLWD)^38^ concerning global surface water distribution. However, the spatial resolution was low, and the level of sub-basins of interest was not achieved. Consequently, we crossed the sub-basins of our study with the Global Surface Water (GSW)^39^ dataset using the Google Earth Engine (GEE)^40^ cloud-based platform. According to GLWD, only 46% of basins with measured chemical data sites were annotated as including surface water, whereas using GSW, 99% of basins were considered to include surface water. The GSW consists of global-scale water-surface maps derived from Landsat 7 and Landsat 8 imagery at 30 m spatial resolution. It contains different layers, and we used the maximum extent layer, which included the area under water at some point in the 1984–2015 period. Since this map also contains seawater, it was filtered with the Global Administrative Unit Layers 2015^41^ database, to avoid overestimating the amount of water in coastal basins. No water information was available from the GSW above 78°N and, consequently GLWD information was used for these Northern Hemisphere high latitudes with the lost of resolution.

### Alkalinity and pH upscaling models

To obtain a global distribution of alkalinity and pH in surface waters, we modeled the relationships between the 107,896 pH values and the 50,916 alkalinity data retained after the quality assurance procedure, and the basin characteristics. The alkalinity data followed a markedly skewed distribution and were log-transformed, with negative and zero values coded as 0.1. The number of modified data in this latter case was low, 0.08% of the total. Because the alkalinity buffering capacity is lost chiefly at values < 10 µeq/L, alkalinities close to zero inherently suffer from a high relative error during lab experimental titration; those differences among negative alkalinity values are usually quite noisy. In any case, a vast proportion of the dataset was far from those very low values, so there was no artificial compression of the distribution, and more cases of very low values could be considered.

A random forest procedure was applied for each variable using the randomForest R package (version 4.7.1.2)^42^. The predictor variables included land surface runoff, elevation, terrain slope, snow cover extent, forest cover extent, annual average air temperature, distance to the most downstream sink, total upstream area, and the percentage of the lithological categories at the upstream watershed. We set the number of decision trees to 200 based on the model stabilization of the out-of-bag error. Seven variables of 23 were randomly sampled as candidates at each split. The alkalinity and pH grouping (Table 4) were used to stratify sampling within each tree to obtain unbiased bootstrap samples.

**Table 4:** RMSE for bootstrapping categories in the out-of-bag and the independent split sets.

	Alkalinity (meq/L)						
Range	<0.2	0.2–0.5	0.5–1	1–2	2–3	3–4	>4
RMSE_OOB_	0.276	0.273	0.352	0.461	0.540	0.638	2.40
RMSE_indep_	0.295 ± 0.05	0.312 ± 0.01	0.399 ± 0.02	0.504 ± 0.01	0.584 ± 0.01	0.709 ± 0.03	2.172 ± 0.43
	**pH**						
Range	<6	6–6.5	6.5–7	7–7.5	7.5–8	8–8.5	>8.5
RMSE_OOB_	0.6	0.3	0.3	0.2	0.2	0.2	0.5
RMSE_indep_	0.7 ± 0.02	0.4 ± 0.01	0.3 ± 0.01	0.3 ± 0.01	0.2 ± 0.01	0.2 ± 0.01	0.6 ± 0.02

The default variance explained by the random forest models was 75% for alkalinity and 64% for pH. For basins with more than one data site, median alkalinity and pH were calculated for validation as reference values, since the model used the median as its site reference and predicted it accordingly. We evaluated model performance using both out-of-bag (OOB) predictions from the random forest, which provide an internal estimate of explained variance, and predictions on independent test data obtained via repeated random 70-30 training-test splits with five random seeds. Root mean square error (RMSE) was calculated for the total out-of-bag set and for the within-group range used in the bootstrapping (Table 4). A general RMSE of 1.12 meq/L for alkalinity and 0.28 for pH was obtained. However, the general alkalinity RMSE was only marginally representative of overall accuracy, as it was significantly inflated by errors at high alkalinity values (Table 4). The distribution of errors across most of the range was quite acceptable, the relative error being lower for intermediate values (Table 4). The errors did not show a significant bias with the predicted values. The R^2^ obtained was 72% for alkalinity and 88% for pH. Using the independent test sets method, the predictive accuracy, averaged across the five iterations, was: for alkalinity, RMSE = 1.06 ± 0.17 meq/L and R² = 74 ± 5.86%; and for pH, RMSE = 0.31 ± 0.0016 and R² = 85 ± 0.15%. A similar pattern across the categories used in the bootstrapping was observed (Table 4).

**Fig. 3:**
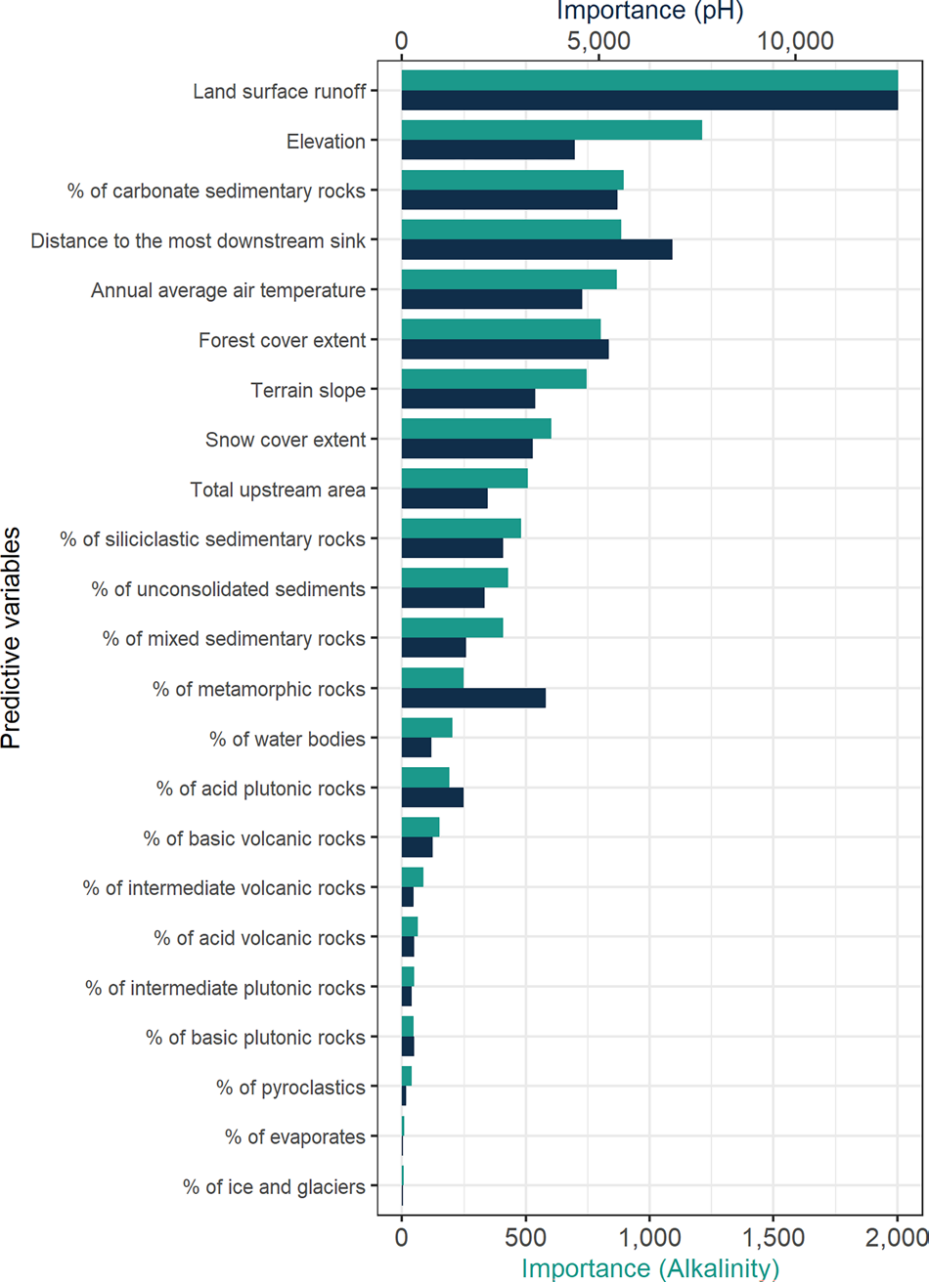
Importance ranks of predictor variables in the random forest models for alkalinity and pH. Note the two different scales for pH and alkalinity to facilitate comparison.

**Fig. 4:**
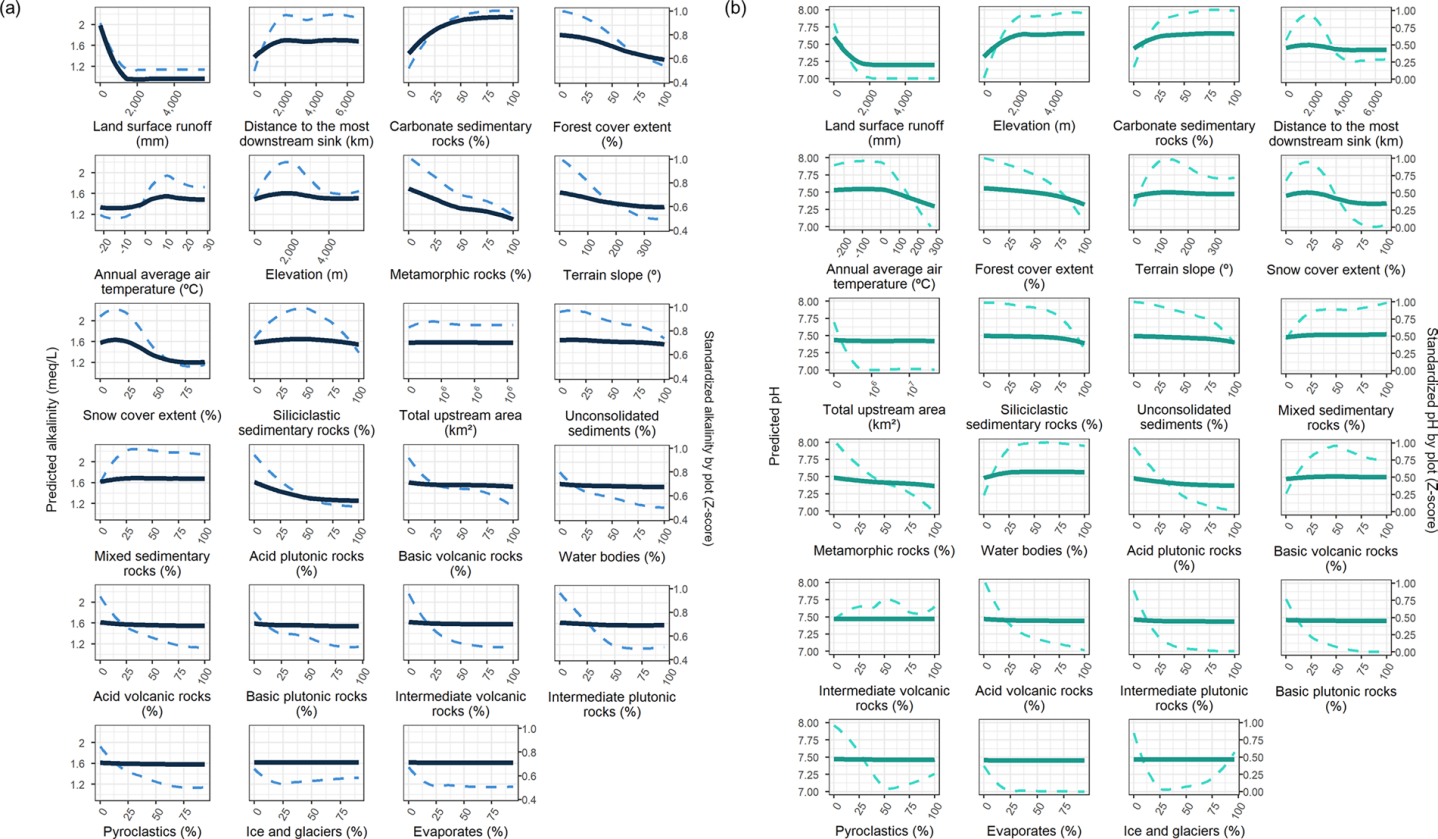
Partial dependence plots of the marginal effect of each predictor variable in the random forest alkalinity (**a**) and pH (**b**) models. The left axis and continuous line indicate absolute values, and the right axis and dashed line indicate standardized values to better visualize variables with smaller effects.

The relevance of each predictive variable was similar in the pH and alkalinity random forest models (Fig. 3). Land surface runoff was the main driver of variation in pH and alkalinity. The other influencing variables were the elevation, proportion of carbonate sedimentary rocks, distance to the most downstream sink, forest cover extent, and average annual air temperature. Nevertheless, there were some differences between pH and alkalinity. Elevation had a greater influence on pH, the second driver, than on alkalinity, the sixth. Distance to the most downstream sink and the percentage of carbonate sedimentary rocks had a greater influence on alkalinity than on pH. The most relevant lithological category was sedimentary rocks (mainly carbonated, but also unconsolidated, mixed, and siliclastic), with the others playing a minor role compared to other factors. Partial dependence plots illustrate the marginal effects of each predictor variable on the alkalinity and pH models (Fig. 4). They show which variables have increasing or decreasing effects, often with nonlinear influences that are particularly significant within specific ranges of the predictor variable.

**Fig. 5:**
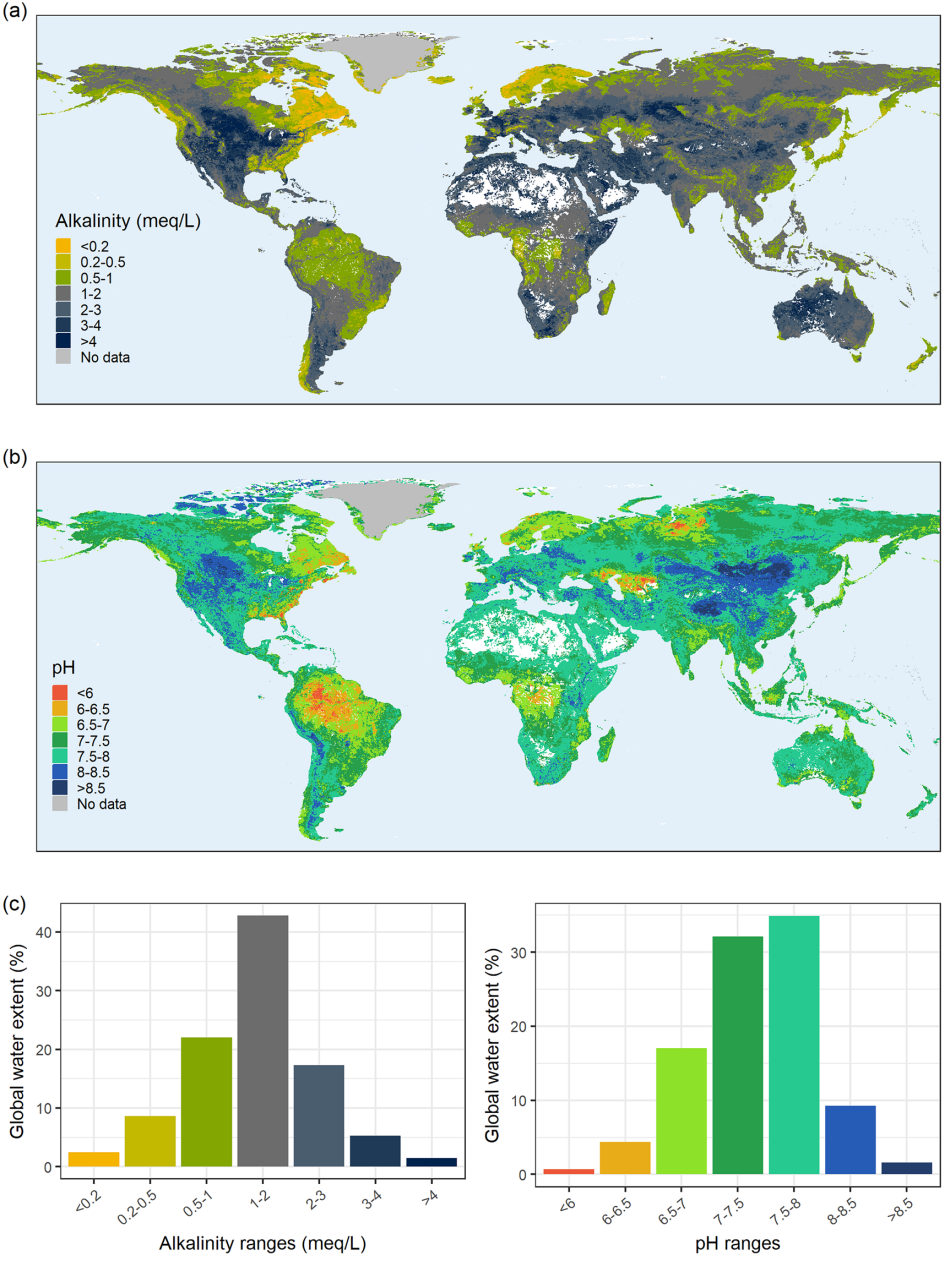
Global distribution of alkalinity (**a**) and pH (**b**); desert basins without surface water are plotted in white. (**c**) Relative water extent across alkalinity and pH gradients.

The upscaling model provided a global mapping of alkalinity and pH in surface inland waters, showing contrasting water-chemistry regions within continents (Fig. 5a,b). Based on the available surface water data for each basin, the relative frequency of alkalinity and pH values in aquatic habitats could be estimated (Fig. 5c). Despite some remarkable regions of low alkalinity and pH, mostly in Northern high latitudes and the tropics, the inland surface waters are largely well-buffered with pH between 7 and 8.

#### Upscaling uncertainty assessment

Field measurements are inevitably biased towards regions of the world with greater monitoring and scientific activity (Table 5). A critical aspect is whether the geographical bias compromises upscaling. In favor of the extrapolation effort was the use of only environmental variables related to purely biogeochemical processes determining water alkalinity and pH. The fundamental mechanisms may vary in intensity but are essentially the same worldwide. These data were available for each drainage basin. Nevertheless, this did not guarantee that the random forest captures all relevant combinations with the existing calibration data, and extrapolation beyond the calibration set’s casuistic may increase uncertainty in data-sparse regions. Therefore, we considered two complementary aspects to assess uncertainty in the upscaling process: (1) the presence of reference analogues, that is, basins with similar environmental conditions in the reference data set; and (2) an indicator of upscaling uncertainty based on the random forest bootstrapping procedure.

**Table 5:** Distribution of measurements and predictions among continents.

Continent	Total basins	Predicted basins with surface water	Basins with pH measurements	Basins with alkalinity measurements
Africa	230,387	116,768	1,711	1,583
Asia	241,656	208,780	2,676	1,714
Europe	175,968	170,553	5,007	1,860
North America	185,122	162,433	32,235	21,901
Oceania	65,851	52,279	728	399
South America	135,148	115,759	3,184	611

Because there were no gaps in the driving variables for the extrapolation, we could evaluate, for each basin, how common the combination of driving values was in the calibration dataset and, thus, whether there were geographical patterns in terms of poor analogues. We quantified the presence of environmental analogues in the reference data set for each basin by calculating the k-nearest neighbor Euclidean distances for the standardized predictor variables^43^. For each basin, we refer to this metric as the environmental differentiation. Larger values imply that a basin lies in regions of the predictor space that are less represented in the training set (i.e., a higher risk of extrapolation bias). Since the variables were previously standardized (i.e., z-scores), the environmental differentiation units are in standard deviations. To mitigate the adverse effects of high dimensionality, as Euclidean distance tends to increase with the number of predictors and become less meaningful^44^, we reduced the number of predictor variables from the initial 23 to the 10 most important in the respective random forest models.

**Fig. 6:**
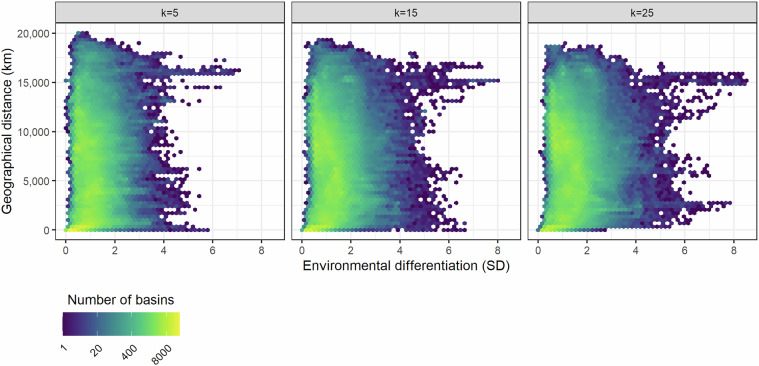
Environmental differentiation, in standard deviation units, compared to geographical distance, considering a different number of k-nearest neighbors. Although there is a highly significant relationship between them due to the large number of sites that are relatively spatially close between them (p-value < 2 × 10^−16^ in all cases), the environmental differentiation variance is poorly explained by the geographical distance (k = 5: R² = 0.00411; for k = 15: R² adjusted = 0.000176; and k = 25, R² = 0.000168).

The environmental differentiation was significantly related to the geographical distance between the basin considered and its k-nearest neighbors. However, the variance explained was markedly low (Fig. 6), around or below 1‰ (R² adjusted in Fig. 6). The relationship vanished at mid and large scales, after a few hundred kilometers. The environmental differentiation barely changed with the number (k) of nearest neighbors considered (Fig. 6), indicating that an environmental analogue in the reference dataset, which is relatively geographically close, exists in most cases.

**Fig. 7:**
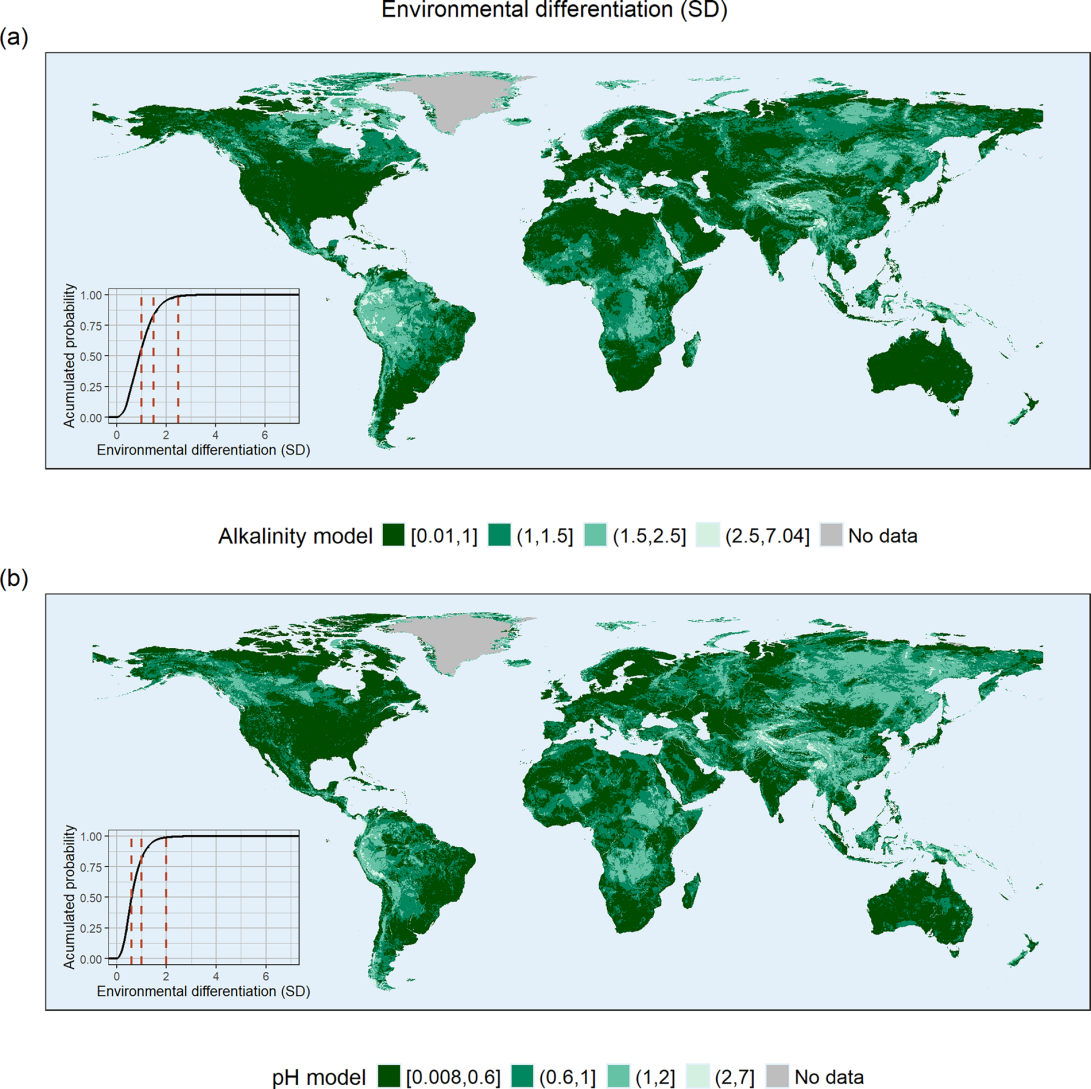
Environmental differentiation for each drainage basin to the reference datasets of alkalinity (**a**) or pH (**b**), obtained considering the 5-nearest neighbors. The distance is in standard deviation units. Four categories were derived from cut-offs at the 50th, 85th, and 99th percentiles, as shown in the insets, respectively indicating highly similar, similar, moderately different, and highly different basins, and illustrated using a declining gradient of greens.

We used k = 5 for the final reporting of each basin environmental differentiation in the published dataset and other comparisons. Based on the distribution of its values, we categorized the environmental differentiation using cut-offs at 50th, 85th, and 99th percentiles, yielding four categories: highly similar basins, similar basins, moderately different basins, and highly different basins (Fig. 7). The categories differed between pH and alkalinity because each variable had a distinct set of the 10 most important predictors and a different number of basins in the reference dataset.

**Fig. 8:**
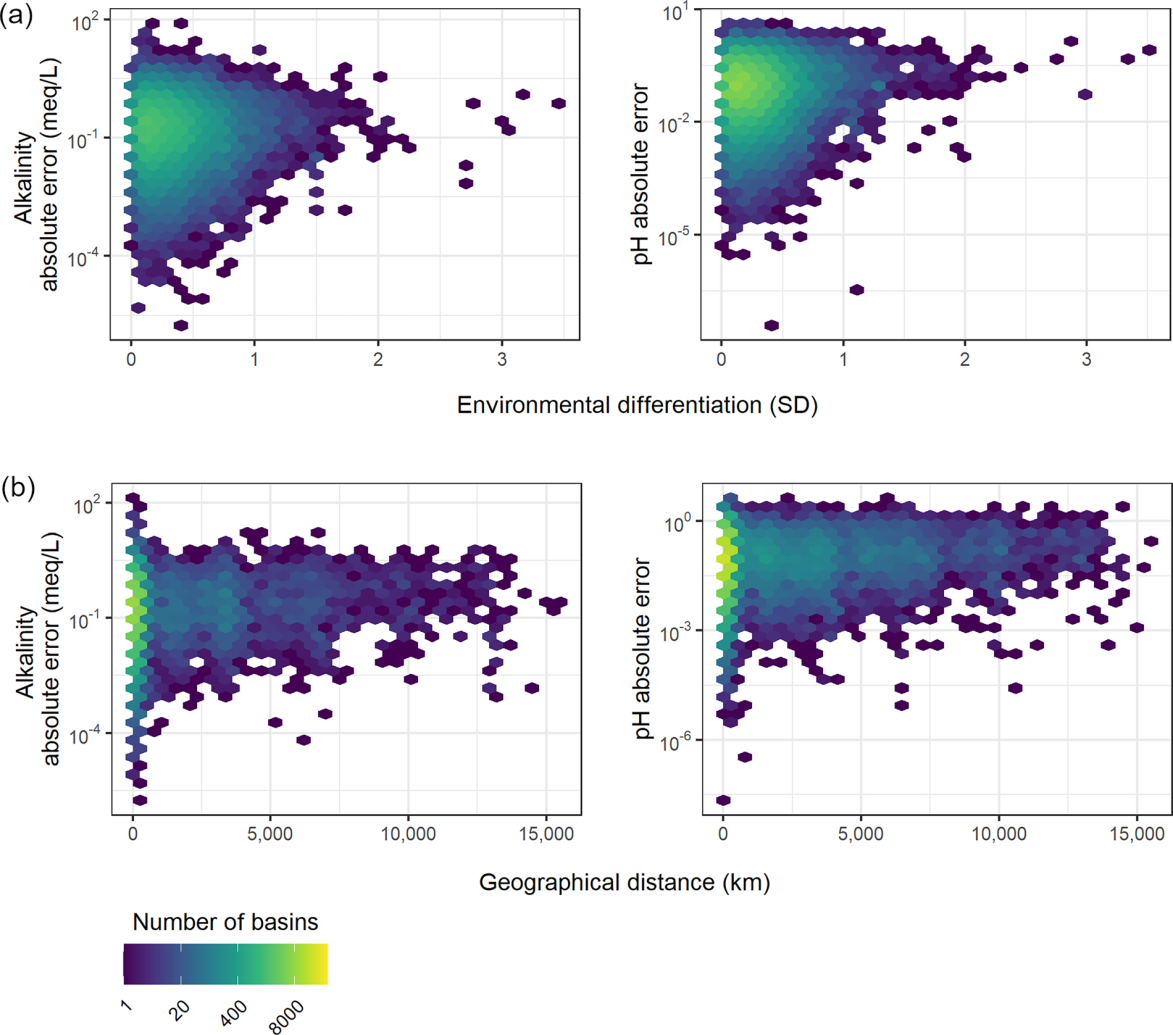
Alkalinity and pH absolute errors from the random forest modelling compared to environmental differentiation (**a**) and geographical distance (**b**).

The difference between modelled and measured data for the reference basins showed, in absolute terms, weak relationships with environmental differentiation and geographical distance (Fig. 8). Absolute errors for alkalinity were not significantly related to environmental differentiation (R^2^ = 1.7 × 10^−5^, p-value = 0.349). For pH, the relationship was significant but had limited predictive capacity, with very low variance explained (R^2^ = 0.00223, p-value = <2 × 10^−16^). Geographic distance showed a significant relationship with alkalinity (R^2^ = 0.00216, p-value = <2 × 10^−16^) and pH (R^2^ = 0.0039, p-value = <2 × 10^−16^), but again with a very low level of variance explained. Across all relationships, the absolute error distributions were asymmetric, with longer tails at lower absolute errors (Fig. 8). The asymmetry was more pronounced for the geographical distance, where the highest error values remained constant across distances, reinforcing the interpretation that environmental analogues have a stronger influence than geographical location in the prediction success.

**Fig. 9:**
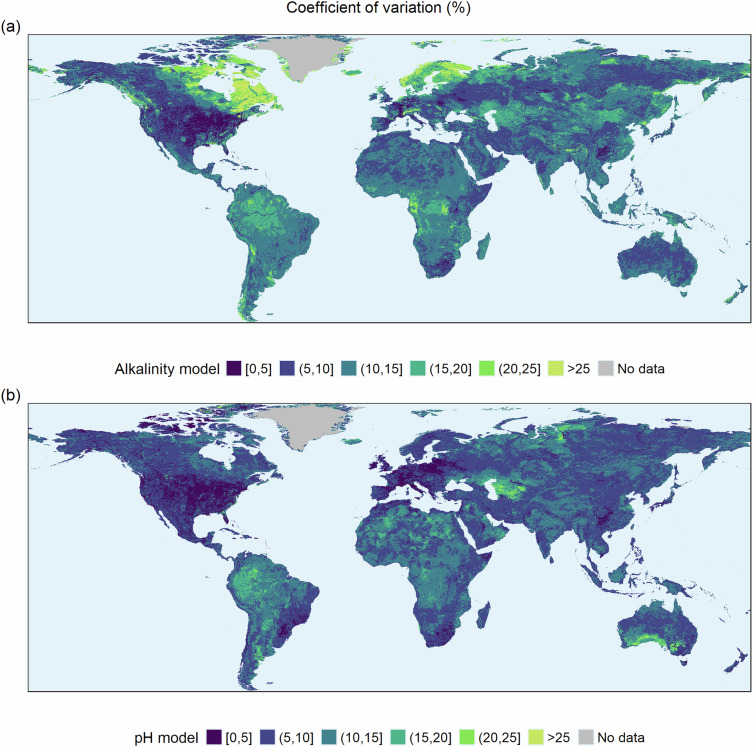
Upscaling confidence indicator for alkalinity (**a**) and pH (**b**) based on the coefficient of variation of the internal bootstrapping process of the random forest. The categories are established to better visualize geographical variation. In the case of alkalinity, uncertainty is higher at low alkalinity values, whereas in the pH case, it appears more closely related to field data density.

The comparison between modelled and measured data allowed evaluating the random forest’s performance with respect to environmental differentiation and geographical distance. However, it did not provide a specific confidence indicator for each final upscaled value. The random forest includes an internal bootstrapping procedure, so changes of the extrapolated values for a given site during the internal process can be recorded, and their variability can be treated as an indicator of uncertainty. Specifically, we calculated the coefficient of variation (%) of the predicted pH and alkalinity values across the 200 trees performed during the procedure as an indicator of upscaling confidence (Fig. 9). Higher values reflected greater relative uncertainty in the predictions. For alkalinity, the geographical distribution of the uncertainty showed increasing relative errors at extreme alkalinity values, particularly in the low-value range, such as in Scandinavia and Northeast Canada. In contrast, the greater availability of pH field measurements led to confidence values more closely linked to field data density in the areas than to the range of values.

In summary, we provide two complementary uncertainty indicators for each basin extrapolation: one related to the degree of analogy of the driving variables in the calibration set, and another to the variation in the model’s output obtained via bootstrapping in the random forest. Both indicators are included in the final dataset along with the upscaled data.

## Data Records

The PHALK dataset is publicly available at the Zenodo repository^45^ under CC BY 4.0 license, using the following structure of files:

- **alkalinity_SurfaceWaters.csv**: Compiled site alkalinity data after quality assurance selection, with the associated predictor variables used in this work and the HydroBASINS v1^20^ polygon code where the site is located. It contains the site coordinates and source references.
- **pH_SurfaceWaters.csv:** Compiled site pH data with the associated predictor variables obtained for this project and the HydroBASINS v1^20^ polygon code where the site is located. It contains the site coordinates and source references.
- **references_SurfaceWaters.csv**: Complete references of the data sources. The ‘reference’ field links records across the alkalinity_SurfaceWaters.csv and pH_SurfaceWaters.csv files via the ‘site_reference’ field.
- **worldwide_estimated_pH_and_alkalinity_at_HydroBasins_HYBASID.csv**: Final pH and alkalinity worldwide estimated values for HydroBASINS^20^ polygons with the associated predictor variables obtained for this project. We also provide the number of sites used for the model within the basin and a field with “1” when it deals with a basin with water surface according to Global Surface Water (or Global Lakes and Wetlands Database if above latitude 78° N), and “0” if no surface water in the basin is assumed. The uncertainty associated with the model was also quantified using the coefficient of variation of the estimated pH and alkalinity values across the 200 bootstrap random forest trees, as well as the environmental differentiation relative to the pH and alkalinity training dataset.
- **files_description.pdf**: Description of all the fields within each file.

If predictor variables were not generated within this work, they are not provided. They can be obtained using the HydroBASINS ID code from https://www.hydrosheds.org/hydroatlas.

The data collation and analyses in this study are based on datasets released under different open licenses, including the Open Government License v3.0 (https://www.nationalarchives.gov.uk/doc/open-government-licence/version/3/), the MIT License (https://opensource.org/licenses/MIT), the Open Government License – Nova Scotia (https://support.novascotia.ca/services/open-data-portal-licence), the Open Government License – Canada (https://open.canada.ca/en/open-government-licence-canada), and Creative Commons licenses that allow redistribution (https://creativecommons.org/licenses/). All sources are openly available, and we comply with the data-sharing terms and conditions.

## Technical Validation

Extensive data validation was conducted across several stages of the process. They have been documented previously in the methodology section because they are naturally embedded in the overall process. Summarizing, they included (1) an initial harmonization of the data from the multiple data sources, (2) the detection of duplicates corresponding to the same sites coming from different sources, (3) the ionic balance analysis for alkalinity quality assurance, and (4) the bootstrapping procedure to evaluate the random forest worldwide upscaling of the alkalinity and pH distribution at HydroBASINS^20^ sub-basin level 12 scale.

## Data Availability

The PHALK dataset is publicly available at the Zenodo repository^45^ (10.5281/zenodo.15591825) under CC BY 4.0 license, using the following structure of files: **• alkalinity_SurfaceWaters.csv**: Compiled site alkalinity data after quality assurance selection, with the associated predictor variables used in this work and the HydroBASINS v1^20^ polygon code where the site is located. It contains the site coordinates and source references. **• pH_SurfaceWaters.csv:** Compiled site pH data with the associated predictor variables obtained for this project and the HydroBASINS v1^20^ polygon code where the site is located. It contains the site coordinates and source references. **• references_SurfaceWaters.csv**: Complete references of the data sources. The ‘reference’ field links records across the alkalinity_SurfaceWaters.csv and pH_SurfaceWaters.csv files via the ‘site_reference’ field. **• worldwide_estimated_pH_and_alkalinity_at_HydroBasins_HYBASID.csv**: Final pH and alkalinity worldwide estimated values for HydroBASINS^20^ polygons with the associated predictor variables obtained for this project. We also provide the number of sites used for the model within the basin and a field with “1” when it deals with a basin with water surface according to Global Surface Water (or Global Lakes and Wetlands Database if above latitude 78° N), and “0” if no surface water in the basin is assumed. The uncertainty associated with the model was also quantified using the coefficient of variation of the estimated pH and alkalinity values across the 200 bootstrap random forest trees, as well as the environmental differentiation relative to the pH and alkalinity training dataset. **• files_description.pdf**: Description of all the fields within each file. We cannot guarantee the total accuracy of the data from the collated databases, although extensive data validation was performed to identify and eliminate errors. The dataset is made available “as is” and is used here under the entire responsibility of the authors.

## Code availability

PHALK dataset preparation was carried out in two main stages. (1) Preprocessing was conducted using Python 3.10.7, based on the code developed in Virro *et al*.^12^. The key packages used were pandas 1.4.2, geopandas 0.10.2, numpy 1.22.2, scikit-learn 0.22, and pyproj 3.3.0. 2). (2) The alkalinity quality analysis was performed using R version 4.2.1. A custom function was developed and made publicly accessible on GitHub (https://github.com/geca-lab/ionic_balance_control), and the Bayesian approach was implemented using the naivebayes 1.0.0. R package.

Spatial analyses and geospatial data visualization were performed using QGIS version 3.26.3^46^ and MiraMon Geographic Information System version 9.0e^47^. Specifically, they were used to extract drainage basin variables. GLiM information was extracted by combining it with sub-basin boundaries using the *CombiCap* module from Miramon. Greenland’s watershed slopes were calculated using the *Slope* tool from the Python GDAL^48^ terrain analysis module (GDAL 3.5.1), and the mean slope per basin was extracted by overlaying the slope raster with the sub-basin boundaries using the *Zonal Statistics* tool, both within QGIS.

We used the Google Earth Engine (GEE) cloud platform to characterize the Global Surface Water (GSW) sub-basins. The code is available on GitHub (https://github.com/geca-lab/global_mapping_pH_and_alkalinity).

Finally, data analysis and alkalinity-pH upscaling models were performed using R version 4.2.1. applying the randomForest package (version 4.7.1.2). For partial dependence analyses, we used the pdp R package (version 0.8.2). Variance inflation factor assessment was performed using the car R package (version 3.1.-3). The get.knnx function from the FNN R package (version 1.1.4.1) was used to compute the environmental differentiation. The model implementation and validation script can be found on GitHub (https://github.com/geca-lab/global_mapping_pH_and_alkalinity).
